# The yin–yang effects of immunity: From monoclonal gammopathy of undetermined significance to multiple myeloma

**DOI:** 10.3389/fimmu.2022.925266

**Published:** 2022-07-25

**Authors:** Zhigang Yi, Tao Ma, Jia Liu, Wenting Tie, Yanhong Li, Jun Bai, Lijuan Li, Liansheng Zhang

**Affiliations:** ^1^ Department of Hematology, Lanzhou University Second Hospital, Lanzhou, China; ^2^ Department of Pediatric Orthopedics and Pediatrics Lanzhou University Second Hospital, Lanzhou, China; ^3^ Department of Hematology, The Affiliated Hospital of Southwest Medical University, Luzhou, China

**Keywords:** multiple myeloma, immune microenvironment, yin–yang, immune cells, immune molecules

## Abstract

Multiple myeloma (MM) is the third most common malignant neoplasm of the hematological system. It often develops from monoclonal gammopathy of undetermined significance (MGUS) and smoldering multiple myeloma (SMM) precursor states. In this process, the immune microenvironment interacts with the MM cells to exert yin and yang effects, promoting tumor progression on the one hand and inhibiting it on the other. Despite significant therapeutic advances, MM remains incurable, and the main reason for this may be related to the complex and variable immune microenvironment. Therefore, it is crucial to investigate the dynamic relationship between the immune microenvironment and tumors, to elucidate the molecular mechanisms of different factors in the microenvironment, and to develop novel therapeutic agents targeting the immune microenvironment of MM. In this paper, we review the latest research progress and describe the dual influences of the immune microenvironment on the development and progression of MM from the perspective of immune cells and molecules.

## Introduction

Multiple myeloma (MM) is a hematological neoplasm with abnormal proliferation of clonal plasma cells (PCs), resulting in the production of large amounts of monoclonal M proteins (1). Common symptoms of MM include impairment of myeloma-related organ function manifestations such as “CRAB” symptoms (hypercalcemia, renal insufficiency, anemia, and bone lesions) and secondary amyloidosis (2). MM accounts for approximately 1% of total malignant tumors and is the third most common hematological malignancy after lymphoma and leukemia, with an estimated 176,404 new cases and 117,077 new deaths in 2020 (3). The median age at diagnosis was approximately 66–70 years in the majority of patients, with 37% of them under 65 years of age (4). In recent years, with the widespread use of new chemotherapy drugs, immunotherapy and autologous stem cell transplantation (ASCT), median survival has significantly improved (5, 6). However, most of the patients remain largely incurable and even suffer from relapse, drug resistance, and death, which brings great burden to the patients and the society. Therefore, treatment still faces huge challenges. It is extremely urgent to study the mechanism of the occurrence and development of MM, improve the cure rate of patients, and minimize the disease recurrence and drug resistance.

As we all know, MM has two important biological characteristics. One is that genetics is highly volatile (7). There are a series of genetic events in the progression of MGUS, SMM to active MM. Initially, post-germinal center B cells are subjected to a series of primary genetic events that progressively progress to MGUS, mainly immunoglobulin heavy chain (IGH) translocations [t(11;14), t(4;14), t(6;14), t(14;16), t(14;20)] and hyperdiploidy (chromosomes 3, 5, 7, 9, 11, 21). Compared to other translocation subgroups, t(14;16) and t(14;20) had significantly higher number of mutations. Moreover, apolipoprotein B mRNA-editing enzyme catalytic polypeptide-like (APOBEC)-related mutations in t(14;16) and t(14;20) translocation groups were significantly higher than other translocation groups (8). APOBEC mutations cause DNA damage and promote genomic instability in MM (9). Conversely, DNA damage closely associated with inflammation may cause abnormal expression of APOBEC family enzymes and altered DNA methylation, leading to altered hematopoietic gene expression (10). As the disease progresses, MGUS clones gain a clonal advantage after being struck by secondary genetic events (such as KRAS mutations, NRAS mutations, and TP53 deletion) and stimulated by drug treatment pressure, and continue to evolve more competitive clones that further drive disease progression. The eventual transformation from inert to aggressive tumor may be an internal factor for MM recurrence, drug resistance, and refractory disease (11–14). Second, the occurrence and development of MM highly depended on the immune microenvironment; these interactions between MM cells and the immune microenvironment, including direct contact and indirect promotion through matrix molecules or various cytokines, lead to MM cell proliferation (15, 16). Thus, approximately 1% of patients with MGUS and 10% of patients with SMM will develop active MM every year (17, 18). Studies have found that abnormalities in the immune microenvironment possibly participate in or even determine the disease progression of MGUS to MM (19). In addition, this interaction forms an immunosuppressive microenvironment, leading to the body’s inability to remove minimal residual disease (MRD) after treatment, which is an external factor for MM recurrence, drug resistance, and refractory (20). However, the immune microenvironment serves different purposes, exerting a tumor cell suppressive effect on the one hand and promoting tumor progression on the other. This role is similar to the yin and yang effects in Chinese traditional medicine. In this review, we update the yin-yang effects of immunity from MGUS to MM, so as to provide a basis for more accurate targeted therapy.

## The yin and yang effects of multiple myeloma progression

**Figure 1:**
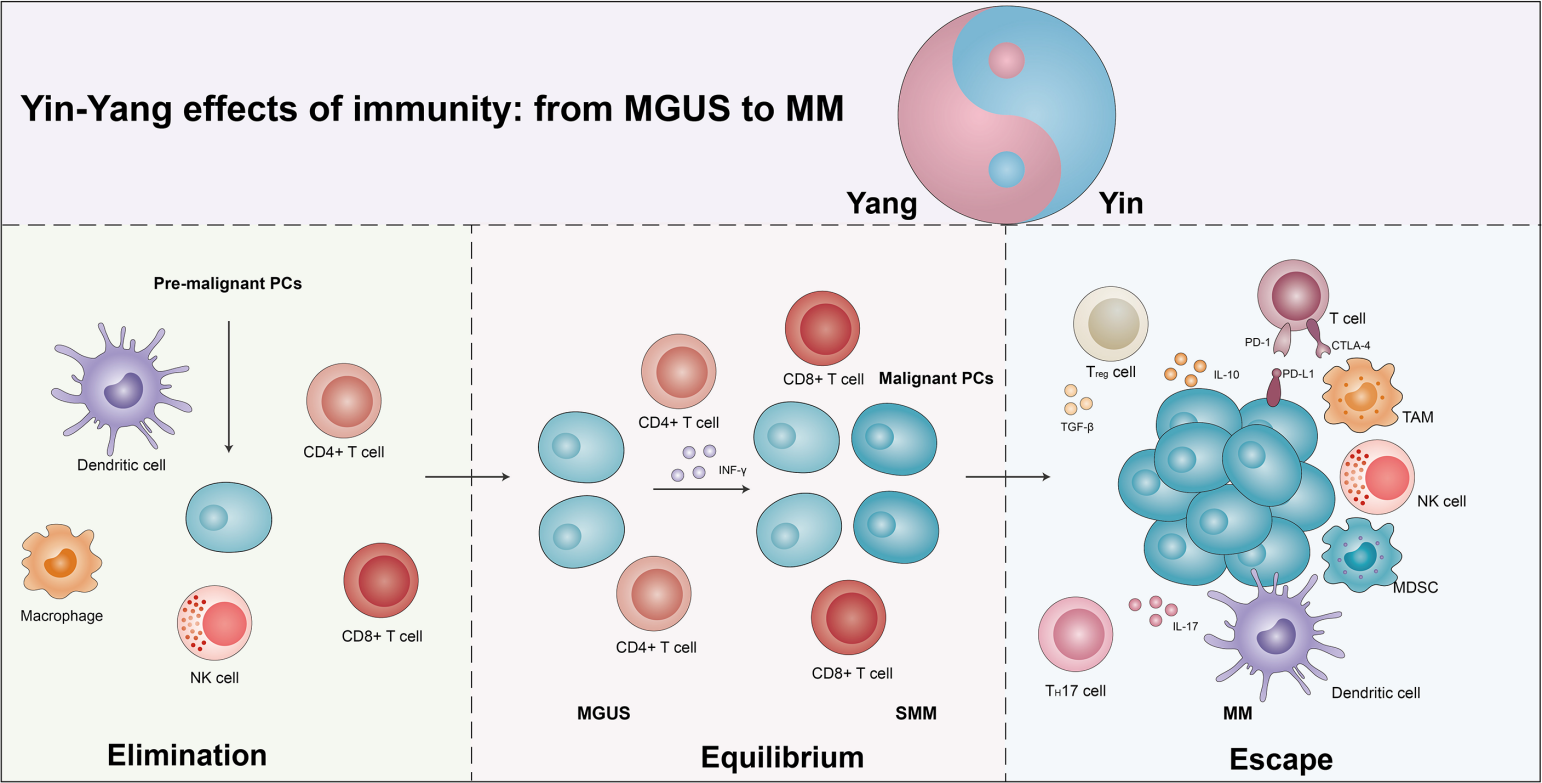
The yin and yang effects of immunity from MGUS to MM. As the body produces pre-malignant plasma cells (PCs), the immune system is activated and eliminated by the immune cells in a process known as the elimination phase, which manifests itself as a yang effect. However, if the elimination is incomplete, some of the pre-malignant PCs become malignant and the immune system enters an equilibrium phase with the tumor cells, which manifests clinically as MGUS/SMM. Due to the continuous immune stress selection, the tumor cells mutate and induce the tumor microenvironment into an immunosuppressive state, leading to the escape phase of the tumor cells. Exhaustion T cells, regulatory T cells (Tregs), suppressor dendritic cells (DCs), dysfunctional NK cells, T helper 17 (Th17) cells, tumor-associated macrophages (TAMs), and bone marrow-derived suppressor cells (MDSCs) promote escape and disease progression, manifesting as a yin effects. See text for detailed explanation.

With the in-depth study of the tumor immune mechanisms, the theory of immunoediting was formally proposed by Schreiber (21, 22). Immunoediting is divided into three stages, immune elimination, immune equilibrium, and immune escape. During immune elimination stage, the body quickly eliminates tumor cells before the tumor appears clinically symptomatic. However, if the mutation of tumor cells is not eliminated in the eradication stage, a few malignant cells are likely to escape the eradication and enter the immune equilibrium stage, during which the malignant cells and immune systems shape each other but the body does not show clinical symptoms. Persistent immune pressure selection results in tumor cells mutating in a state of genetic instability and imbalance that is no longer recognized by adaptive immunity and insensitivity to antitumor immune effector mechanisms, inducing tumor microenvironment into an immunosuppressive state and resulting in tumor cells entering the escape stage, where tumor growth is no longer blocked by the immune system, thus presenting immune tolerance (23). Studies have found that similar to solid tumors, the MM progression also occurs throughout the immunoediting processes (**Figure 1**) (24, 25).

Increasing studies suggest that MGUS/SMM may be representative of immune equilibrium and subsequent disruption of equilibrium during the progression in MM (25, 26). A single-cell RNA sequencing revealed an increase in the quantity of NK cells, T cells, CD16+ cells, and non-classical monocytes, and a decreased number of plasmacytoid dendritic cells (pDCs), immature neutrophils, and CD14+ monocytes in the MGUS stage. Several of the alterations have already been observed in the early stages of MGUS. Meanwhile, the accumulation of regulatory T cells (Tregs) and γδT cells was observed, with a subsequent loss of CD8+ memory populations and elevated IFN signaling in the SMM stage. Conversely, they found that MM cells caused a loss of antigen presentation and induced T cells’ suppressor phenotype (16). The CD8+ memory T cells play an important role in tumor immunity (27). In MM, CD8+ central memory T cells were moderately reduced, while there was a marginally higher ratio of CD8+ effector/effector memory T cells. T-cell factor 1 (TCF1) expression levels were significantly elevated in memory CD8+ T cells from MGUS patients, while no alterations were observed in the expression of T-bet, EOMES, and GATA-3. In addition, the percentages of TCF1hi cells were obviously elevated in MGUS patients while TCF1^-^ cells were elevated in the MM group. In MGUS and MM, the most obvious differences in T cells are related to two different T-cell types of clusters (T2 and T3). In the T2 cluster, there was an over-representation of MGUS and an under-representation of MM, and the expression of stem-like genes (TCF1/TCF7) was notably increased. The T3 cluster enriched in MM significantly increased the expression of KLRG1 (senescence-associated gene), PRDM1 (a marker of exhaustion), and Fos, and downregulated granulysin and lysozyme (28).

Immune checkpoints are a class of immunosuppressive molecules whose high expression causes depletion of T cells, thereby reducing immune surveillance and killing of tumor cells, and eventually lead to immune evasion of tumor cells. The programmed cell death protein (PD)-1/PD-ligand (PD-L1) axis, the most representative immune checkpoint, controls the antitumor immune response to solid tumors and malignant hematologic diseases (29). Federica et al. found that in comparison to MGUS patients, PD-L1 expression was elevated in CD138+ MM cells in both MM and SMM patients. Moreover, there was an inversion of the CD4+/CD8+ ratio in patients with relapsed MM, followed by increased levels of IL-6 expression. There was a remarkable positive correlation between %CD14+PD-L1+ and %CD8+PD-1+ cells in relapsed patients compared to the patients with SMM and newly diagnosed MM (NDMM) (30). Therefore, MGUS and MM obviously exhibit the immune yin-yang effects.

Host-associated immunodeficiency contributes to the development of MM from MGUS/SMM (31). The depletion in peripheral blood (PB) B cells and the upregulation in T cells were found in MM progression. This alteration of immune status is strictly related to immune paralysis during the progression of MM. They also observed the same trends in B, T, and NK cells in SMM non-progressors versus SMM progressor patients. This variation specifically shows that SMM progressor patients reduced the proportion of CD57 lymphocyte subsets (including the CD57-CD16+ and CD57-CD56+). Moreover, the expression of PD-L1 in CD138+ MM cells was higher compared to MGUS and SMM patients. The above signs indicate a state of immune depletion and exhaustion during the progression of MM.

The tumor immune microenvironment favored angiogenesis and related to the progression of MM from asymptomatic to symptomatic, with poor prognosis and therapy resistance (32). In MM Vk*MYC mice, microvessel density (MVD) was almost twice that of SMM mice, and highly correlated with the level of monoclonal antibodies in the blood. Two cytokines for angiogenesis [vascular endothelial growth factor (VEGF-A) and IL-18] were significantly increased in Vk*MYC mice at the stage of MM (33). Meanwhile, in Vk*MYC mice bearing oncogene-driven PC proliferative barriers, immune microenvironment changed, including progressively decreased T helper (Th)1 and continuously increased Th2 cytokine secretion, which related to the accumulation of CD206CTie2C macrophages. Therefore, angiogenesis in the tumor immune microenvironment also performs a critical role in the progression of tumor cells.

## The yin and yang effects of immune cells associated with multiple myeloma progression

### Innate immune cells

#### Dendritic cells

Dendritic cells (DCs) are extensively presented antigen-presenting cells (APCs) that efficiently uptake, process, and deliver antigens and have the ability to promote the activation and differentiation of naive T lymphocytes (34, 35). Generally, DCs are broadly divided into two major types: plasmacytoid DCs (pDCs) and myeloid DCs (mDCs). Results regarding the number, phenotypic status, and function of DCs are controversial during progression from MGUS to MM. Compared to healthy donors (HDs), pDCs were significantly reduced in MM PB and bone marrow (BM) patients. More importantly, the prominently reduced pDCs were also found in MGUS *vs*. MM patients. A similar study has found that the proportion of both mDCs and pDCs are decreased from MGUS/SMM to MM (36). In addition, the frequency of mDCs and pDCs is negatively associated with disease progression in MM patients (37). Another research has reported that DCs from MM patients are functionally impaired, although they are numerically normal. They cannot increase the expression of CD80 (B7-1) after huCD40LT stimulation because they are inhibited by transforming growth factor-β1 (TGF-β1) and IL-10 (38). Moreover, the migration and secretion of IL 12p70 and interferon-gamma (IFN-γ) were significantly reduced in MM-DCs (39). On the contrary, Patrizia et al. found that mDCs and pDCs accumulated in the BM during the progression of MGUS to MM. After phagocytosis of apoptotic tumor PCs by CD91, BM-mDCs and pDCs can stimulate the activation of tumor-specific CD8+ T cells. By interacting directly with CD28 on non-apoptotic tumor PCs, BM-mDCs downregulate the proteasomal subunit expression in these cells, thereby preventing them from being killed by human leukocyte antigen (HLA) class I-limited CD8+ T cells (40).

**Figure 2:**
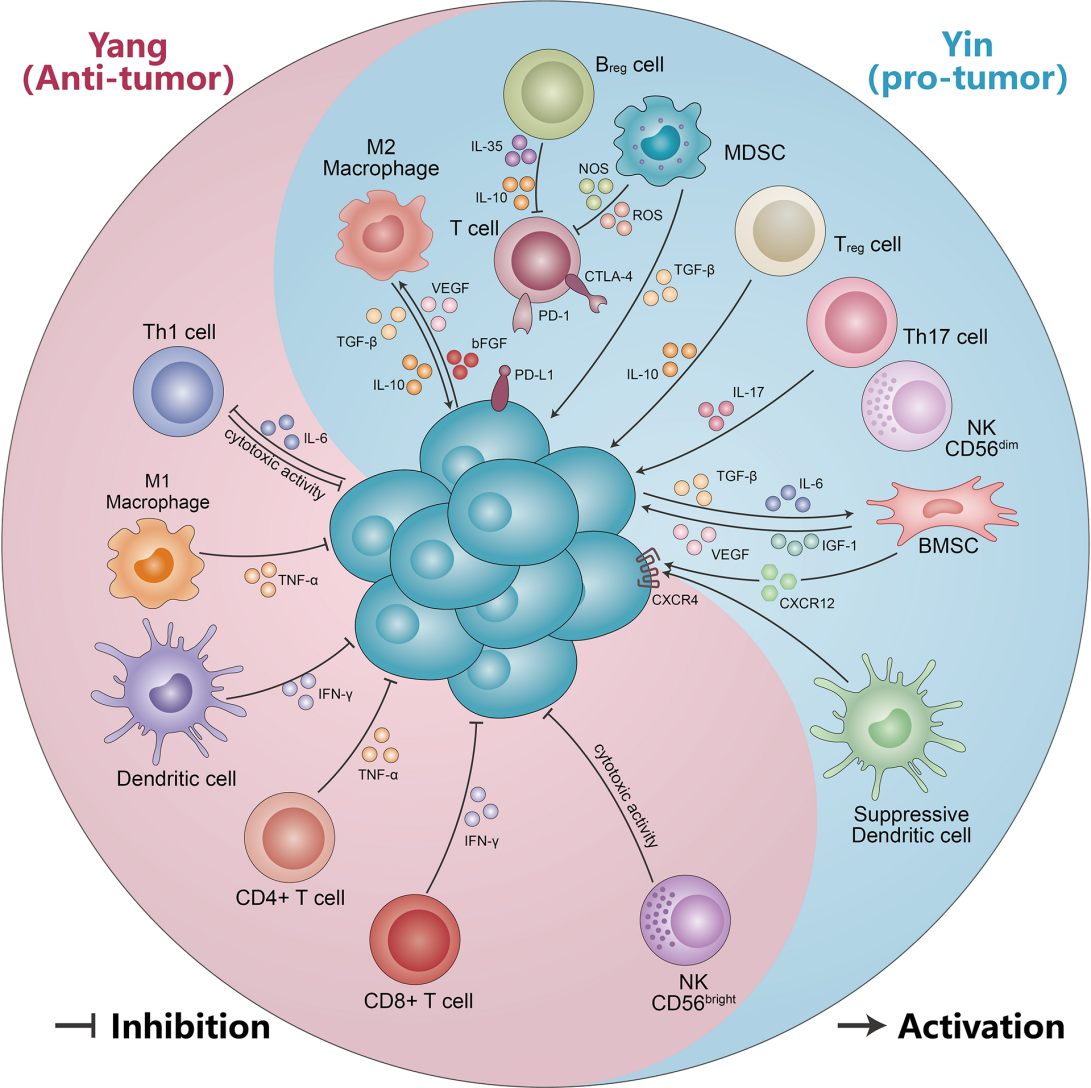
The yin and yang effects of immunity cells and non-cellular components. Immune cells (including CD56^bright^ NK cells, DC cells, CD4+ T cells, CD8+ T cells, M1-like macrophages, and Th1 cells) suppress malignant PCs by secreting IFN-γ, TNF-α, and cytotoxic effects. Meanwhile, malignant PCs can form an immunosuppressive microenvironment (including M2-like macrophages, MDSC, Treg cells, Th17 cells, suppressor DC cells, CD56^dim^NK cells, and Bregs) by secreting IL-10, TGF-β, and IL-6. The immunosuppressive microenvironment in turn can be suppressed by VEGF, IL-17, IGF-1, IL-10, and TGF-β, which promotes multiple myeloma progression. See text for detailed explanation.

In addition, DCs’ surface expression of maturation markers and stimulation of allogeneic proliferation were aberrant. There was a marked trend towards lower expression of HLA-DR and HLA-A, B, and C on mDCs and pDCs of MM patients compared to HDs (41). Similar to the expression of C-C chemokine receptor 5 (CCR5) and CCR7, expression was also decreased in mDCs and pDCs from MM patients compared with HDs. Conversely, the expression of CD86 and CD83 showed an elevated trend. At same time, the ability of DCs to stimulate the proliferation of CD4+ and CD8+ T cells is impaired in MM patients. After chemotherapy and ASCT, HLA-DR and HLA-A, B, and C expression in mDCs and pDCs was higher than in patients with MM at diagnosis (41). In conclusion, the yin and yang effects of DCs in the progression of MM are obviously distinct (**Figure 2**, **Table 1**).

**Table 1:** Yin and yang effects of immune cells associated with multiple myeloma progression.

Immune cells	Effects in different stages of the disease			Refs.
	MGUS/SMM		MM	
DCs	Yang (Antitumor)	Yin (Pro-tumor): suppressive		33–38
TAMs	Yang: M1 macrophages, promoting the destruction of tumor cells, recruiting tumor-killing leukocytes, or directly phagocytosing tumor cells	Yin: M2 macrophages, promoting tumor cell proliferation, distant metastasis, and suppressing immunity		43–50
NKs	Yang (Antitumor)	Yin (Pro-tumor)		13, 51–58
MDSCs	–	Yin (Pro-tumor)		59–67
CD4+/CD8+ T	Increased: Yang (Antitumor)	Decreased: Yin (Pro-tumor)		68–70
Tregs	–	Yin (Pro-tumor)		71–75
Th17	–	Yin (Pro-tumor)		76–80
Bregs	–	Yin (Pro-tumor)		81–84

DCs: dendritic cells, TAMs: tumor-associated macrophages, NKs: natural killer (NK) cells, MDSCs: myeloid-derived suppressor cells, Tregs: regulatory T cells, Th17: T helper 17 cells, Breg: regulatory B cells.

#### Tumor-associated macrophages

Tumor-associated macrophages (TAMs) are the most enriched immune cells in the tumor immune microenvironment, which are derived from circulating monocytes and tissue-resident macrophages (TRMs) (42, 43). Activated macrophages are classified into two different types: M1 and M2. M1 macrophages are activated by IFN-γ, lipopolysaccharide (LPS), and granulocyte macrophage-colony stimulating factor (GM-CSF) and then secrete IFN-γ, IL-6, IL-12, tumor necrosis factor-alpha (TNF-α), reactive oxygen species (ROS), and nitric oxide synthase (NOS), exhibiting pro-inflammatory features such as promoting the destruction of tumor cells, recruiting tumor-killing leukocytes, or directly phagocytosing tumor cells (44). On the contrary, M2 macrophages promote tumor cell proliferation, distant metastasis, drug resistance, and angiogenesis and suppress immunity, which is stimulated by IL-10, transforming growth factor beta (TGF-β), and neovascularization agents VEGF and fibroblast growth factor-2 (FGF-2) (45). M2 macrophages express high levels of CD206, CD163, and TGFβR, while M1 macrophages express high levels of CD40, CD80, and CD86 (43). Both M1 and M2 macrophages are highly plastic and can be interconverted in response to changes in the tumor microenvironment or therapeutic intervention.

In recent years, a large amount of research evidence has demonstrated that macrophages have an essential role in the progression of MM, such as promoting BM PC homing and proliferation, angiogenesis, and angiogenic mimicry (46–49). Macrophages in the blood effectively supported the proliferation of MM cell lines through contact-mediated and non-contact-mediated mechanisms, and contributed to the *in vitro* growth of primary CD138+ cells in the BM of MM patients. Importantly, co-culture with macrophages protects MM from chemotherapeutic drug-induced cell death and significantly promotes IL-1β, chemokine C-C motif ligand-2 (CCL2), CCL5, and IL-8 expression in MM cells at the mRNA level. Moreover, MM cells educate macrophages and promote M2 polarization (50). In the BM of MM patients, CD163+CD206+ M2 macrophages were significantly increased compared with SMM and MGUS patients. The function, phenotype, and morphology of active MM were distinct from patients with stable disease and MGUS (50). Furthermore, the study found that overall survival (OS) was obviously shorter in patients with CD68+ macrophages (85). Wang et al. showed that patients with higher CD163+ M2 macrophage expression at MM diagnosis had worse progression-free survival (PFS) and OS, and achieved lower rates of complete remission (CR)/near-CR rate, particularly relapsed and aggressive MM patients (86).

MM is a highly vascularized tumor, with increased neovascularization leading to tumor progression. CD163+ M2 macrophages were found to be correlated with MVD. In a xenograft mouse model of MM, binding of clodronate liposomes (Clo) to VEGFA siRNA significantly suppresses tumor growth. The expression of angiogenesis and VEGFA expression were obviously higher in the control than in Clo and Clo+ si. In addition, the number of neovascularization upregulated the number of M2 macrophages. CD163+ cells were clearly more numerous in the Clo+ M2 group than in the Clo+ M1 group (87). Scavelli et al. indicated that macrophage expression with VEGF and bFGF obtained endothelial cell (EC) markers when MM is in an active state of disease. Meanwhile, macrophages adapted functionally, phenotypically similar to MM patient-derived endothelial cells (MMECs). This cannot occur in MGUS or benign anemia patients, likely minimal in nonactive MM (46). Thus, neo-angiogenesis and angiogenesis play a vital role in MM progression, supporting the idea that macrophages may promote MM growth by stimulating MM-associated neo-angiogenesis through paracrine secretion. MM-associated macrophages also have the capacity to be directly involved in MM-associated neo-angiogenesis. Therefore, M1-like macrophages have an immune yang effect and M2-like macrophages have a yin effect in the progression of MM (**Figure 2**, **Table 1**).

#### Natural killer cells

Natural killer (NK) cells are a vital component of the innate immunity and have an important role in tumor immunity, especially in hematological tumors (88–91). Unlike T cells, NK cells can directly kill cancer or infected cells without antigenic pre-stimulation, major histocompatibility complex (MHC) class I molecule presentation, and antibody recognition (92). Moreover, it also produces a large number of cytokines, which regulate the adaptive immune responses and are involved in other related pathways (93, 94). NK cells are divided into CD56^bright^ and CD56^dim^ types based on the surface density of CD56 changes, which exhibit different phenotypic characteristics. The CD56^bright^ NK cells can directly produce a large amount of cytokines, while CD56^dim^ NK cells have a stronger cytotoxicity and express significantly more immunoglobulin-like receptors and FcγRIII (Fcγ receptor III, also named CD16) (95). As the first line of defense, NK cells rapidly remove pathogens and tumor cells from the body. The triggering of NK cells depends on two modes of “missing self” and “induced self” (51). In the event of viral infection or cellular carcinogenesis, MHC-I molecules’ expression on the cell surface is either absent or low, resulting in a loss of function of the NK cell surface killer activation receptors by “missing self”. In addition to downregulating MHC class I molecule expression, some neoplasms and virus-infected cells may also combine and reactivate killer activation receptors on the surface of NK cells, which is called “inducing self” (52, 53).

The correlation between NK cells and MM progression remains controversial. Numerous studies have found NK cell dysfunction from MGUS/SMM to MM (**Figure 2**, **Table 1**). A recent single-cell RNA sequencing study reveals that NK cell abundance is frequently increased in patients with MGUS, associated with a more immature NK cell subpopulation and subsequent phenotypic shift in MM progression, suggesting a possible compromised immune system. Furthermore, they also observed that the NK cells’ enrichment in MGUS patients had a significant enrichment for the C-X-C motif chemokine receptor (CXCR) 4 CXCR4+ subset, while lower NK cells’ frequencies displayed the low CXCR4 and CX3CR1+ subset (16). Another study found that the SMM and MM patients had higher percentages of CD56^dim^ NK cells in PB compared with HDs, while the relapsed/refractory multiple myeloma (RRMM) and especially post-autologous stem cell transplant (pSCT) patients had obviously lower CD56^dim^ NK cells. By comparison, the CD56^bright^ NK cells of RRMM and pSCT had higher percentages, and this increased accumulation may be the result of NK cell reactivation after previous treatment or chemotherapy drugs and stem cell transplant (SCT) depletion.

In addition, the expression receptors on the surface of NK cells such as CD57, FcγRIII, CD226, NKG2D, SLAM family member 7 (SLAMF7), and natural cytotoxicity receptors (NCRs) have been found to be altered (54). Bernal et al. found that the MM PCs had the highest MHC-I molecules, followed by MGUS PCs and the lowest expression on cells without monoclonal gammopathy. The activated NKG2D ligand MICA followed a reverse order (55). However, Carbone et al. had a different conclusion in that early-stage MM patients express a lower level of MHC-I molecules and higher levels of NKG2D, MICA, and MICB, but an opposite expression level in the late stage (56). Decreased expression of ligands or activated NK cell receptors led to the functional quiescence of NK cells and immune evasion (57). Moreover, several studies revealed that the expressions of 2B4 and DNAM-1 were decreased in MM, but NCRs had no changes (54, 58). The NK cells’ capacity for antibody-dependent cellular cytotoxicity (ADCC) declined, especially in advanced disease (96). This response depended on the expression of activation receptors and the respective ligands on myeloma cells (97). The degranulation response of NK cells could also assess NK cell function. Compared to HDs, MM patients showed significantly decreased expression of the NK cell degranulation marker CD107a. In RRMM and pSCT patients, CD107a expression was lower under ADCC conditions (54). Therewith, the elevated expression of the inhibitory receptor [such as PD-1/PD-L1, T-cell immunoglobulin and ITIM domains (TIGIT)] interacts with the ligand expressed on MM cells and mediates NK cell depletion. Meanwhile, NK cell recovery is achieved until 30 days after autologous hematopoietic stem cell transplantation (auto-HSCT). Importantly, at +30 and +100 days after auto-HSCT, MM patients with a lower frequency of mature, well-differentiated NKG2A-CD57+ NK cell subsets had a better PFS to the next treatment than those with a higher frequency (98). This provides new insights into the importance and degree of differentiation in NK cell reconstitution, which may have a better prognosis of MM patients after auto-HSCT.

#### Myeloid-derived suppressor cells

Myeloid-derived suppressor cells (MDSCs) are a heterogeneous group of BM-derived cells that are precursors to DCs, macrophages, and/or granulocytes (59). Under normal conditions, BM hematopoietic stem cells firstly differentiate into myeloid precursor cells (MPCs), and then rapidly into mature granulocytes, DCs, and macrophages, which enter the appropriate organs to perform immune functions (60). However, in pathological conditions such as tumor infection and inflammation, the maturation of MPCs is hindered by inflammatory factors or tumor-derived cytokines, and they acquire immature and dysfunctional myeloid suppressor cells (MDSCs) (61). There are two major subsets of MDSCs, monocyte-like MDSCs (Mo-MDSCs) and granulocyte-like MDSCs (G-MDSCs) (62). In mice, Mo-MDSCs present a CD11b+ Ly6G- Ly6C^high^ phenotype and G-MDSCs present a CD11b+ Ly6G^high^ Ly6C^low^ phenotype, while in humans, Mo-MDSCs exhibit CD11b+ CD33+ HLA-DR^-/low^ CD14+ and G-MDSCs exhibit CD11b+ CD33+ HLA-DR^-/low^ CD14- (63).

Recently, the roles of MDSCs have been reported in different cancer types, especially MM (64, 65). Several studies have found that MDSCs differ in number function and phenotype in MM patients compared with MGUS patients and HDs. Favaloro et al. discovered an absolute and relative increase in the number of G-MDSCs in both PB and BM in MM patients. Meanwhile, both patients with progressive disease and stable disease had significantly higher proportions of G-MDSCs compared to age-matched controls. Similar to the G-MDSC, patients with progressive disease also had higher BM Mo-MDSC levels than those with NDMM. High Mo-MDSC levels had significantly poorer prognosis than patients with lower Mo-MDSCs. High Mo-MDSC can be used as an important poor prognostic indicator (66). In addition, the MDSC burden is closely related to MM stages, therapeutic response to bortezomib-based treatment, and pool clinical outcome (67). Similar to other solid tumors, MM cells have a bidirectional interaction with other cells of the immune microenvironment: regulating tumor development on the one hand, and transforming the BM microenvironment into an immunosuppressive environment on the other. The phenotype and frequency of MDSCs in BM and PB of patients with NDMM or RRMM were analyzed such that the frequency of MDSCs in RRMM increased with disease progression compared to HDs. The inhibitory molecules reactive species of oxygen (ROS) and arginase-1 (ARG1) significantly increased. More importantly, MM-MDSCs can directly induce MM cell proliferation, and conversely, MM cells can also trigger the development of MDSCs by inhibited activity against autologous T cells. This immunosuppression is manifested by downregulation targeting CD4+ T, CD8+ T, and NKT cell-mediated antitumor immune responses. In addition, neither lenalidomide nor bortezomib changed this effect. Meanwhile, Tregs can also be inhibited (62, 66). Mesenchymal stromal cells (MSCs) have strong immunosuppressive effects. MSCs stimulate the proliferation of MDSCs and suppress their apoptosis. Additionally, MSCs enhanced MDSCs by suppressing T-cell proliferation and IFN-γ production. Furthermore, both the Arg1 and NOS2 mRNA and protein levels were upregulated in MDSCs. These findings demonstrate that MSCs may perform immunomodulatory effects on MDSCs through the upregulation of Arg1 and NOS2 (99).

In MM-bearing mice models, MDSCs accumulate mainly in the spleen and lymph nodes, which promote MM growth. During the progression of MM in the 5TMM mouse model, the accumulation of MDSCs in the BM was observed in the early stages of disease progression, while an increase in circulating myeloid cells was observed in the later stages (100). Another research showed that polymorphonuclear/granulocytic (PMN)-MDSCs displayed a higher suppressive potential and a pro-angiogenic role by the expression and upregulation of vasculogenic-related factors. Interestingly, they observed Mo-MDSCs as osteoclast precursors (68). In summary, MDSCs play a yin immunological role in the progression of MM.

Other myeloid cell lineages also participated in the development of MM, for example, neutrophils in the absolute number between MM, MGUS, and HDs, but they found that neutrophils isolated from MM had a reduced phagocytic activity and an immunosuppressive function of T cells, indicating that neutrophils may contribute to the impairment of MM immune function. Petersson et al. found that BM neutrophils of MM patients exhibited MDSC function (69). However, high-density neutrophils (HDNs) have been found in MM and, to a lesser extent, in MGUS. HDNs from MM have induced the upregulation of FcγRI (also known as CD64) and the downregulation of structural FcγIIIa, as well as decreased phagocytic activity and oxidative burst (70). HDNs may promote MM progression through increased susceptibility to infection and immune dysfunction. Human PB monocytes are a population of heterogeneous cells. They are generally divided into three categories, classical (CD16-CD14+), non-classical (CD16+CD14^dim^), and intermediate (CD16+CD14+) (71). Compared with HDs, the proportion of CD16-CD14+ monocytes was remarkably lower in MM patients, while the proportion of CD16+CD14^dim^ and CD16+CD14+ monocytes was significantly higher. CD16+CD14^dim^ and CD16+CD14+ monocyte ratios were positively correlated with serum PCs, M-protein, calcium, creatinine, and lactate dehydrogenase (LDH) levels and negatively correlated with serum albumin levels. The proportion of CD16-CD14+ monocytes was positively correlated with albumin levels and negatively correlated with serum M-protein, PCs, calcium, creatinine, and LDH levels (72). Sponaas et al. found that as tumor load increases, the quantity of CD16+CD14^dim^ monocytes has been shown to increase (73). Meanwhile, another study found that in MM and SMM patients, PD-L1 was expressed at higher levels in CD14+CD16+ monocytes than CD14+CD16- cells, independent of disease stage (30). Therefore, other types of cells also play an important role in MM progression (**Figure 2**, **Table 1**).

#### Adaptive immune cells T cells

The aberrant function and number of T cells present in the progression of MM. The normal CD4/CD8 T-cell ratio in BM was as follows: age ≤ 1 year [0.9 (0.5–1.2)]; 1 year < age ≤ 4 years [0.5 (0.4–0.6)]; 4 years < age ≤ 15 years [0.4 (0.3–0.6)]; age > 15 years [0.4 (0.3–0.5)] (74). The CD4+/CD8+ T-cell ratio was abnormal in MGUS or MM patients. In untreated myeloma patients, the CD4+ cells were downregulated in both percentage and absolute numbers, while the CD8+ T cells were slightly upregulated (75). Additionally, the CD28, CD152, ZAP-70, and PI3K involved in T-cell signaling and the signal transduction molecules are significantly reduced in CD4+ and CD8+ T cells, especially in the advanced MM stage (101). The decrease in the number of CD4+ cells was associated with the clinical stage, a shorter survival, high β2-microglobulin (β2M), and anemia. Another study found a strong T-cell response to autologous precancerous cells in MGUS patients. This pre-tumor-specific CD4+ and CD8+ T-cell response was detected in T cells freshly isolated from BM. MM-BM-derived T cells are deficient in this tumor-specific fast effector function. This phenomenon could be explained by the fact that the increased tumor burden from MGUS to MM leads to T-cell exhaustion.

Inflammation is one of the characteristics of MM, as it is highly dependent on inflammation during disease progression. A variety of inflammatory molecules are involved in this process, such as IL-6, IL-10, and TGF-β. Meanwhile, MM being an age-related disease, the senescent body has decreased immune cell function and is unable to perform biological functions, leading to a gradual accumulation of senescent cells in the body, causing the body to enter a specific chronic inflammatory state. Not only is inflammation a symptom of senescence, it may also drive the key factors of diseases associated with the aging process (76). During the process of senescence, the expression of pro-inflammatory factors is caused by an imbalance between the innate and acquired immune systems of the body. Their long-term stimulation leads to chronic, low-grade, inflammatory senescence and increases the development of age-related diseases. Multiple signaling pathways are involved in the above processes, such as NF-kB, JNK, and RIG-1 pathways (77). Thus, inflammation and senescence interact in the development of MM and together contribute to the progression of the disease. Zelle-Rieser et al. found that T cells from MM displayed the characteristics of exhaustion and senescence in the tumor area. There was an increased expression of PD1, cytotoxic-T-lymphocyte-antigen-4 (CTLA-4), CD160, and 2B4 on CD8+ T cell from BM of MM patients (78). Importantly, CTLA4 is expressed not only on the surface of T cells, but also on CD19+ B cells, but its expression in the immune microenvironment of MM has not been reported in relevant studies (79). Under continuous antigenic stimulation, the expression of CD28 gradually and irreversibly decreases, while CD57 expression increases, manifesting a state of replicative senescence. In both HDs and MM patients, most of the T cells deleted CD28 expression while CD57 expression was notably upregulated in MM-BM T cells. Thus, compared to HDs’ BM, the total amount of CD57+ CD28- CD8+ T cells was obviously increased. Furthermore, after therapy with immunomodulatory drugs and dexamethasone, the proportion of senescent CD57+CD28− CD8+ T cells was reduced (78).

Regulatory T cells (Tregs), which are generated by the thymus and exported to the periphery, inhibit in a positive regulatory manner the activation and proliferation of potentially self-reactive T cells present in the normal body, thereby significantly suppressing immune action. Tregs are clearly classified into thymus-derived tTregs, peripherally induced pTregs *in vivo*, and *in vitro* induced iTregs. CD4+CD25+Foxp3+ is considered to be the classical combination marker for Tregs (80). The proportions of CD4+CD25+ cells were significantly elevated in MGUS and MM patients compared with HDs. The Foxp3 and CTLA4 expression also decreased in MGUS and MM patients. Moreover, Tregs did not inhibit anti-CD3-mediated T-cell proliferation in MGUS or MM patients (102). The local changes are manifested such that the proportion of Tregs is higher in MRD-positive patients than in MRD-negative patients. In a mouse model of MM based on MOPC cells, the BM section showed that Tregs highly accumulated at the site of tumor growth. Tregs from BM MOPC-MM mice expressed higher levels of activation markers of CD25, CD69, and CD44 and inhibitory receptors T-cell immunoglobulin mucin-3 (Tim-3), lymphocyte-activation gene 3 (Lag3), and TIGIT compared with healthy mice. In mice, Treg depletion rapidly leads to the activation of CD8+ T cells and NK cells as major effector cells against MM (103). The evolution from MGUS to MM is related to alterations in Tregs and terminal effector CD8+ T cells (TTE). This may be associated with the expression of CD39 and CD69, influencing the adenosine metabolic pathway and its residence in the BM microenvironment, as well as the oligoclonal expansion of CD8+ TTE cells (104). Conversely, studies also reported an association between the presence of BM-infiltrating regulatory T cells and dysfunctional CD4+PD-1+ cells and inferior survival in NDMM patients (81).

T helper 17 (Th 17) cells are a group of IL-17-secreting T cells that require co-induced differentiation by IL-6 and TGF-β. They play a very important role in host defense, inflammation, and autoimmunity (82). Th17 cells were altered with different therapy stages of MM. The percentage of Th17 cells in peripheral blood mononuclear cells (PBMCs) was significantly increased in NDMM, partial remission (PR), and disease relapse myeloma patients, but significantly decreased in CR (83). Prabhala et al. found that Th17-associated cytokines (such as IL-17, IL-23, and IL-13) were significantly elevated in MM patients compared with HDs. Moreover, IL-17 promotes MM cell growth and suppresses immune function. Downregulation of Th1 cell responses by Th17-secreted cytokines in myeloma. Several studies have identified an abnormal ratio of Th17 and Tregs cells in the progression of MM (84). Remarkable differentiation of Treg/Th17 ratio was observed between normal and MM patients. The absolute number of Th17 cells is elevated and Treg cells are reduced in MM patients, which results in a significant imbalance in the Th17/Treg cell ratio. This change normalizes with disease stabilization (105, 106). In addition to the abnormal Th17/Treg ratio, there were also aberrant Th1 and Th2 ratios. Thus, the yin and yang effects of T cells appear to be particularly pronounced in the pathogenesis of MM (**Figure 2**, **Table 1**) (107).

#### Regulatory B cells

Regulatory B cells (Bregs) are a subset of B lymphocytes that have immunomodulatory functions and maintain immune tolerance. Through secretion of IL-10, IL-35, and TGF-β, Bregs inhibit immunopathology *via* preventing the expansion of pathogenic T cells and other pro-inflammatory lymphocytes (108). Recent studies have demonstrated that the inflammatory environment of different diseases induces different Breg populations (109, 110). In MM, Bregs–myeloma cell interactions enable immunosuppression and promote their survival in the BM environment. In MM, regulatory CD19+CD24^high^CD38^high^ B cells, which have immunosuppressive properties, are more clearly defined in BM than in PB. The proportions of BM-Bregs within CD19+ cells are remarkably increased in NDMM patients compared to patients who responded to treatment (maintenance). However, BM-Bregs from NDMM patients are dramatically reduced 1 day after CD138+ myeloma cell deletion. In CD138-depletion of BM mononuclear cells (BMMCs) (CD138-BM), the frequency of apoptosis BM-Bregs was notably increased as compared to freshly harvested BMMC (BM) and with the addition of CD138+ myeloma cells (111). Zou et al. found that the proportion of CD19+CD24^high^CD38^high^ Bregs within CD19+ B cells significantly differed at different stages of MM. Namely, in MGUS patients, the percentage of CD19+CD24^high^CD38^high^ Bregs was markedly higher compared to MM. In addition, the B-cell percentage in NDMM was positively correlated with Breg percentage. Patients with Bregs < 10% had significantly shorter OS and PFS (112). Another study showed that the proportion of Bregs with CD19+CD24^high^CD38^high^ was higher than in HDs. While the percentage of CD19+CD24^high^CD38^high^ Bregs in MM patients decreased after treatment with daratumumab (113). Furthermore, the Breg combination with PET/CT can predict the therapeutic response and survival in contemporary patients with NDMM (114). Thus, Bregs also display the yin and yang effect in MM (**Figure 2**, **Table 1**).

## The yin and yang effects of non-cellular components associated with multiple myeloma progression

Non-cellular components mainly include cytokines, growth factors, pro-angiogenesis factors, and chemokines. Cytokines, growth factors, pro-angiogenesis factors, and chemokines are secreted into the fluid environment of the BM, and the interaction of MM cells with the BM microenvironment is of paramount importance in the progression of MM (115–117).

IL-6 plays a pathogenetic role in MM and promotes the growth of MM cells (118). The levels of IL-6 in the MM group were higher than those in HDs and associated with Durie-salmon (DS) stages and treatment cycle. Elevated serum IL-6 levels are factors in the poor prognosis of MM patients (119). However, another research revealed that the high expression level of IL-6 is linked to low tumor burden and low proliferation scores in MM (120). Frassanito et al. found that the production of autocrine IL-6 in MM patients paralleled the clinical stage of disease. The highest percentage of IL-6+ cells was detected in resistant relapse or primary refractory patients. Then, in the absence of exogenous IL-6, the MM cells were characterized by a high susceptibility to spontaneous apoptosis (121). Systemic levels of IL-6 may be useful as prognostic factors of MM bone disease (122). BM IL-6 levels in MM patients are highly correlated with bone resorption rates and serum C-terminal telopeptide of collagen I (ICTP) and urinary N-telopeptide (uNTx) (122, 123).

IL-10 is a key anti-inflammatory mediator that protects the host from pathogen and microbiota overreaction, while playing an active role in other environments such as sterile wound healing, autoimmunity, and cancer (124). Serum IL-10 levels were obviously increased in MGUS patients compared to HDs and lower than those observed in MM patients (125). Wang et al. found that high IL-10 levels lead to significantly worse PFS and OS in patients, suggesting that the serum IL-10 levels are a novel predictor of prognosis in MM (126). IL-10 can also induce PC proliferation and angiogenesis in MM. Serum levels of IL-10 correlated positively with VEGF, angiopoietin-2 (Ang-2), B cell-activating factor (BAFF), and infiltration. Furthermore, increased IL-10 expression parallels disease progression and advanced international staging system (ISS) stage (127). Minnie et al. found that CD8+ T cells derived from MM relapsed mice showed high IL-10 secretion, which was related to the increase in the expression of TIGIT and PD-1 (128).

TGF-β is an important modulator of cell growth and differentiation, which has been demonstrated to suppress the proliferation of dormant hematopoietic stem cells and induce the differentiation of late progenitor cells into red blood cells and BM cells (129). TGF-β plays a vital role in hematological malignancies, including leukemia, lymphoma, and MM (130). TGF-β1 is produced in MM by tumor cells and bone marrow mesenchymal stem cells (BMSCs), and associated with tumor cell growth. In addition, the inhibitory effect of tumor cell resistance to TGF-β1 on normal B-cell proliferation and immunoglobulin secretion may have promoted MM cell growth (131). Serum TGF-β1 levels were in the normal range in patients without immunoparesis, whereas they were increased in patients with immunoparesis (132). Thus, patients with higher TGF-β1 levels appeared to have functional immune impairment in MM. TGF-β receptor (TβRIII) expression is reduced or absent in most MM specimens. Functionally, restoration of TβRIII expression in MM cells significantly suppressed cell proliferation and motility, mainly independent of its ligand-presenting action (133). TGF-β also promotes osteolytic bone disease associated with MM. Inhibition of TGF-β activation delays tumor progression and bone destruction in an MM mouse model (134). One possible mechanism is that TGF-β suppression stimulates collagen maturation to increase bone repair and fracture resistance, and another mechanism is that TGF-β suppression can release stromal cells from the differentiation block of MM and induce osteoblast differentiation, which inhibits the proliferation and survival of MM cells, increases the sensitivity of MM cells to chemotherapeutic drugs, and overcomes stromal cell-mediated drug resistance (135, 136).

Angiogenesis plays an essential role in the development of MM. VEGF is a key molecule involved in the angiogenic process of MM. Alexandrakis showed that VEGF was increased in MM patients and was distinctively higher in stage III disease compared to stage I and stage II. In addition, there were positive correlations of VEGF and IL-6, TNF-α, β2M, C-reactive protein (CRP), and LDH (137). Another research found that most of the MM cases exhibited strong VEGF expression. Also, VEGF expression is positively correlated to MVD (138). VEGF and its type 2 receptor (VEGFR2) polymorphisms are related to the increased risk and aggressiveness in MM (139).

Insulin-like growth factor-1 (IGF-1) is a group of factors that promote cell growth and have insulin-like metabolic effects. In recent years, its role in the regulation of normal and malignant hematopoietic growth has received increasing attention (140, 141). IGF-1 promotes vascular endothelial cell and BM stromal cell lineage trafficking. The mechanism is through activation of PI3K/PKC and PI3K/RhoA pathways independent of Akt to promote myeloma cell migration (142). Peng et al. demonstrated that the acquisition of mesenchymal characteristics is enhanced by IGF-1 in a time-dependent manner. *In vitro* studies showed that the IGF-1-mediated mesenchymal phenotype contributes to the migration, invasion, and colony formation of MM. Mechanistic studies suggested that IGF-1 induces epithelial–mesenchymal transition (EMT) in MM cells by the PI3K/Akt signaling pathway (143). IGF-1 is also a growth and survival factor in MM cell lines (144). In clinical trials, Standal et al. found that serum IGF-1 levels were not different between MM and healthy age- and sex-matched controls. Nevertheless, MM patients with low IGF-1 level had not reached median survival (145). This suggests that IGF-1 is a prognostic factor.

The C-X-C motif chemokine ligand 12 (CXCL12) is also called stromal cell-derived factor-1 (SDF-1), which is selectively overexpressed in several tissues and organs, and functions as the ligand for C-X-C motif chemokine receptor 4 (CXCR4). The CXCR12/CXCR4 axis has emerged as a potential therapeutic target through the activation of multiple signaling pathways, such as ERK1/2, Ras, p38 MAPK, PLC/MAPK, SAPK/JNK, and regulation tumor stem cells, which play a vital role in tumor initiation and progression (146, 147). Their antagonists have been generated and show encouraging results in terms of anti-cancer activity. The level of CXCR4 expression was increased in BM of MM patients compared to HDs (148). On the contrary, several studies found that CXCR4 expression was inversely correlated with disease status and survival of MM patients. Patients with active MM exhibited a significantly lower expression of CXCR4 compared to those with inactive disease (149). CXCR4 is a good prognostic indicator of survival for MM patients (150). MM PCs produce significant levels of SDF-1 protein and shows higher level of expression compared with normal subjects, and elevated serum SDF-1 levels are associated with an increased osteoclast activity, bone destruction, and tumor angiogenesis in MM patients (151, 152). Tumor PCs also increased CX43 expression in MSCs, and led to an elevated CXCL12 expression and stimulated CXCR4 expressed on MM cells. The resulting CX43/CXCL12/CXCR4 interaction boosted mitochondrial trafficking in MSCs and protected tumor cells from the effects of anti-myeloma drugs (153). Furthermore, blockade of the SDF-1/CXCR4 axis reduces adhesion-mediated resistance to chemotherapy in MM cells through interaction with IL-6 (154). The CXCR4-specific inhibitor AMD3100 and the antibody against CXCR4 MAB171 inhibit MM cell migration *in vitro*. The CXCR4 knockout assay showed that SDF-1-dependent migration was mediated through PI3K and ERK/MAPK pathways, but not p38 MAPK (155). Moreover, MM cells recruit tumor-supporting macrophages by the CXCR4/CXCL12 axis and drive their polarization towards the M2 phenotype (50). In a murine model, injection of RPMI-8226 caused an osteolytic lesion proximal to the tumor, leading to a 5% reduction in bone volume (BV) compared with control. Importantly, systemic application of the CXCL12/CXCR4 antagonist T140 significantly inhibited bone loss (156). Thus, different immune molecules play different yin and yang roles in the development of MM (**Figure 2**).

## Conclusion

The immune microenvironment is critical to the development and progression of MM. In recent years, with the rapid development of immunotherapy, researchers have begun to focus on the role of the immune microenvironment in the pathogenesis and treatment of MM, with the expectation that new therapeutic targets will be identified. In this article, we provide a comprehensive overview on how the immune microenvironment regulates the development of MM, both in its negative role of promoting the immune escape of tumor cells and in its positive role of limiting tumor growth through the activation of antitumor immunity. However, the immune microenvironment is a dynamic and complex process, which is one of the root causes of MM recurrence and refractory to treatment. At the same time, we are faced with the question of when to use immunotherapy, for which patients, and how to use more efficacious immune-targeted therapies. Therefore, a more precise understanding of the interactions between MM and the immune microenvironment will help provide a scientific basis for better immunotherapy. In the future, as research continues to progress, we believe that increasingly precise immunotherapy approaches will emerge to achieve maximum survival time for MM patients.

## Author contributions

All authors contributed to the article and approved the submitted version.

## Funding

This work was supported by a commissioned Project of National Clinical Medicine Research Center for Hematological System Diseases (2021WWA01), the Talent Innovation and Entrepreneurship Project of Lanzhou (2020‐RC‐48), the Cuiying Scientific and Technological Innovation Program of Lanzhou University Second Hospital (CY2017‐ZD04, CY2019‐MS14, and CY2021-QN-A12), and the Lanzhou University Education Development Foundation scientific research project [(21)0854].

## Conflict of interest

The authors declare that the research was conducted in the absence of any commercial or financial relationships that could be construed as a potential conflict of interest.

## Publisher’s note

All claims expressed in this article are solely those of the authors and do not necessarily represent those of their affiliated organizations, or those of the publisher, the editors and the reviewers. Any product that may be evaluated in this article, or claim that may be made by its manufacturer, is not guaranteed or endorsed by the publisher.

## References

[1] Kumar SK Rajkumar V Kyle RA van Duin M Sonneveld P Mateos MV. Multiple myeloma. Nat Rev Dis Primers (2017) 3:17046. doi: 10.1038/nrdp.2017.46 28726797

[2] Rajkumar SV Dimopoulos MA Palumbo A Blade J Merlini G Mateos MV. International myeloma working group updated criteria for the diagnosis of multiple myeloma. Lancet Oncol (2014) 15(12):e538–548. doi: 10.1016/S1470-2045(14)70442-5 25439696

[3] Sung H Ferlay J Siegel RL Laversanne M Soerjomataram I Jemal A. Global cancer statistics 2020: GLOBOCAN estimates of incidence and mortality worldwide for 36 cancers in 185 countries. CA Cancer J Clin (2021) 71(3):209–49. doi: 10.3322/caac.21660 33538338

[4] Kazandjian D. Multiple myeloma epidemiology and survival: A unique malignancy. Semin Oncol (2016) 43(6):676–81. doi: 10.1053/j.seminoncol.2016.11.004 PMC5283695 28061985

[5] Mikkilineni L Kochenderfer JN. CAR T cell therapies for patients with multiple myeloma. Nat Rev Clin Oncol (2021) 18(2):71–84. doi: 10.1038/s41571-020-0427-6 32978608

[6] Rajkumar SV. Multiple myeloma: 2020 update on diagnosis, risk-stratification and management. Am J Hematol (2020) 95(5):548–67. doi: 10.1002/ajh.25791 32212178

[7] Furukawa Y Kikuchi J. Molecular basis of clonal evolution in multiple myeloma. Int J Hematol (2020) 111(4):496–511. doi: 10.1007/s12185-020-02829-6 32026210

[8] Walker BA Wardell CP Murison A Boyle EM Begum DB Dahir NM. APOBEC family mutational signatures are associated with poor prognosis translocations in multiple myeloma. Nat Commun (2015) 6:6997. doi: 10.1038/ncomms7997 25904160 PMC4568299

[9] Talluri S Samur MK Buon L Kumar S Potluri LB Shi J. Dysregulated APOBEC3G causes DNA damage and promotes genomic instability in multiple myeloma. Blood Cancer J (2021) 11(10):166. doi: 10.1038/s41408-021-00554-9 34625538 PMC8501035

[10] Olinski R Styczynski J Olinska E Gackowski D. Viral infection-oxidative stress/DNA damage-aberrant DNA methylation: separate or interrelated events responsible for genetic instability and childhood ALL development? Biochim Biophys Acta (2014) 1846(1):226–31. doi: 10.1016/j.bbcan.2014.06.004 25003587

[11] van Nieuwenhuijzen N Spaan I Raymakers R Peperzak V. From MGUS to multiple myeloma, a paradigm for clonal evolution of premalignant cells. Cancer Res (2018) 78(10):2449–56. doi: 10.1158/0008-5472.CAN-17-3115 29703720

[12] Melchor L Brioli A Wardell CP Murison A Potter NE Kaiser MF. Single-cell genetic analysis reveals the composition of initiating clones and phylogenetic patterns of branching and parallel evolution in myeloma. Leukemia (2014) 28(8):1705–15. doi: 10.1038/leu.2014.13 24480973

[13] Pawlyn C Morgan GJ. Evolutionary biology of high-risk multiple myeloma. Nat Rev Cancer (2017) 17(9):543–56. doi: 10.1038/nrc.2017.63 28835722

[14] Keats JJ Chesi M Egan JB Garbitt VM Palmer SE Braggio E. Clonal competition with alternating dominance in multiple myeloma. Blood (2012) 120(5):1067–76. doi: 10.1182/blood-2012-01-405985 PMC3412330 22498740

[15] Kawano Y Roccaro AM Ghobrial IM Azzi J. Multiple myeloma and the immune microenvironment. Curr Cancer Drug Targets (2017) 17(9):806–18. doi: 10.2174/1568009617666170214102301 28201978

[16] Zavidij O Haradhvala NJ Mouhieddine TH Sklavenitis-Pistofidis R Cai S Reidy M. Single-cell RNA sequencing reveals compromised immune microenvironment in precursor stages of multiple myeloma. Nat Cancer (2020) 1(5):493–506. doi: 10.1038/s43018-020-0053-3 33409501 PMC7785110

[17] Landgren O Kyle RA Pfeiffer RM Katzmann JA Caporaso NE Hayes RB. Monoclonal gammopathy of undetermined significance (MGUS) consistently precedes multiple myeloma: a prospective study. Blood (2009) 113(22):5412–7. doi: 10.1182/blood-2008-12-194241 PMC2689042 19179464

[18] Kyle RA Remstein ED Therneau TM Dispenzieri A Kurtin PJ Hodnefield JM. Clinical course and prognosis of smoldering (asymptomatic) multiple myeloma. N Engl J Med (2007) 356(25):2582–90. doi: 10.1056/NEJMoa070389 17582068

[19] Manier S Sacco A Leleu X Ghobrial IM Roccaro AM. Bone marrow microenvironment in multiple myeloma progression. J BioMed Biotechnol (2012) 2012:157496. doi: 10.1155/2012/157496 23093834 PMC3471001

[20] Quach H. MRD end point in myeloma: ready for prime time? Blood (2022) 139(6):799–802. doi: 10.1182/blood.2021013363 35142848

[21] Schreiber RD Old LJ Smyth MJ. Cancer immunoediting: integrating immunity's roles in cancer suppression and promotion. Science (2011) 331(6024):1565–70. doi: 10.1126/science.1203486 21436444

[22] Dunn GP Bruce AT Ikeda H Old LJ Schreiber RD. Cancer immunoediting: from immunosurveillance to tumor escape. Nat Immunol (2002) 3(11):991–8. doi: 10.1038/ni1102-991 12407406

[23] Dirnhofer S Zippelius A. Cancer immunology, inflammation, and tolerance: an introduction. Virchows Arch (2019) 474(4):405–6. doi: 10.1007/s00428-019-02547-3 30796517

[24] Nakamura K Smyth MJ Martinet L. Cancer immunoediting and immune dysregulation in multiple myeloma. Blood (2020) 136(24):2731–40. doi: 10.1182/blood.2020006540 32645135

[25] Korde N Kristinsson SY Landgren O. Monoclonal gammopathy of undetermined significance (MGUS) and smoldering multiple myeloma (SMM): novel biological insights and development of early treatment strategies. Blood (2011) 117(21):5573–81. doi: 10.1182/blood-2011-01-270140 PMC3316455 21441462

[26] McCachren SS Dhodapkar KM Dhodapkar MV. Co-Evolution of immune response in multiple myeloma: Implications for immune prevention. Front Immunol (2021) 12:632564. doi: 10.3389/fimmu.2021.632564 33717170 PMC7952530

[27] Ando M Ito M Srirat T Kondo T Yoshimura A. Memory T cell, exhaustion, and tumor immunity. Immunol Med (2020) 43(1):1–9. doi: 10.1080/25785826.2019.1698261 31822213

[28] Bailur JK McCachren SS Doxie DB Shrestha M Pendleton K Nooka AK. Early alterations in stem-like/resident T cells, innate and myeloid cells in the bone marrow in preneoplastic gammopathy. JCI Insight (2019) 5. doi: 10.1172/jci.insight.127807 PMC6629164 31013254

[29] Armand P. Immune checkpoint blockade in hematologic malignancies. Blood (2015) 125(22):3393–400. doi: 10.1182/blood-2015-02-567453 25833961

[30] Costa F Vescovini R Marchica V Storti P Notarfranchi L Dalla Palma B. PD-L1/PD-1 pattern of expression within the bone marrow immune microenvironment in smoldering myeloma and active multiple myeloma patients. Front Immunol (2020) 11:613007. doi: 10.3389/fimmu.2020.613007 33488620 PMC7820813

[31] Dosani T Mailankody S Korde N Manasanch E Bhutani M Tageja N. Host-related immunodeficiency in the development of multiple myeloma. Leuk Lymphoma (2018) 59(5):1127–32. doi: 10.1080/10428194.2017.1361026 PMC6750254 28792255

[32] Ribatti D Vacca A. New insights in anti-angiogenesis in multiple myeloma. Int J Mol Sci (2018) 19(7). doi: 10.3390/ijms19072031 PMC6073492 30002349

[33] Calcinotto A Ponzoni M Ria R Grioni M Cattaneo E Villa I. Modifications of the mouse bone marrow microenvironment favor angiogenesis and correlate with disease progression from asymptomatic to symptomatic multiple myeloma. Oncoimmunology (2015) 4(6):e1008850. doi: 10.1080/2162402X.2015.1008850 26155424 PMC4485787

[34] Mellman I Steinman RM. Dendritic cells: specialized and regulated antigen processing machines. Cell (2001) 106(3):255–8. doi: 10.1016/S0092-8674(01)00449-4 11509172

[35] Weinstock M Rosenblatt J Avigan D. Dendritic cell therapies for hematologic malignancies. Mol Ther Methods Clin Dev (2017) 5:66–75. doi: 10.1016/j.omtm.2017.03.004 28480306 PMC5415319

[36] Knight A Rihova L Kralova R Penka M Adam Z Pour L. Plasmacytoid dendritic cells in patients with MGUS and multiple myeloma. J Clin Med (2021) 10(16). doi: 10.3390/jcm10163717 PMC8396926 34442012

[37] Pasiarski M Grywalska E Kosmaczewska A Gozdz S Rolinski J. The frequency of myeloid and lymphoid dendritic cells in multiple myeloma patients is inversely correlated with disease progression. Postepy Hig Med Dosw (Online) (2013) 67:926–32. doi: 10.5604/17322693.1065871 24018459

[38] Brown RD Pope B Murray A Esdale W Sze DM Gibson J. Dendritic cells from patients with myeloma are numerically normal but functionally defective as they fail to up-regulate CD80 (B7-1) expression after huCD40LT stimulation because of inhibition by transforming growth factor-beta1 and interleukin-10. Blood (2001) 98(10):2992–8. doi: 10.1182/blood.V98.10.2992 11698282

[39] Shinde P Fernandes S Melinkeri S Kale V Limaye L. Compromised functionality of monocyte-derived dendritic cells in multiple myeloma patients may limit their use in cancer immunotherapy. Sci Rep (2018) 8(1):5705. doi: 10.1038/s41598-018-23943-w 29632307 PMC5890285

[40] Leone P Berardi S Frassanito MA Ria R De Re V Cicco S. Dendritic cells accumulate in the bone marrow of myeloma patients where they protect tumor plasma cells from CD8+ T-cell killing. Blood (2015) 126(12):1443–51. doi: 10.1182/blood-2015-01-623975 PMC4592278 26185130

[41] Brimnes MK Svane IM Johnsen HE. Impaired functionality and phenotypic profile of dendritic cells from patients with multiple myeloma. Clin Exp Immunol (2006) 144(1):76–84. doi: 10.1111/j.1365-2249.2006.03037.x 16542368 PMC1809645

[42] Cheng N Bai X Shu Y Ahmad O Shen P. Targeting tumor-associated macrophages as an antitumor strategy. Biochem Pharmacol (2021) 183:114354. doi: 10.1016/j.bcp.2020.114354 33279498

[43] Hoeffel G Ginhoux F. Ontogeny of tissue-resident macrophages. Front Immunol (2015) 6:486. doi: 10.3389/fimmu.2015.00486 26441990 PMC4585135

[44] Pan Y Yu Y Wang X Zhang T. Tumor-associated macrophages in tumor immunity. Front Immunol (2020) 11:583084. doi: 10.3389/fimmu.2020.583084 33365025 PMC7751482

[45] Krneta T Gillgrass A Poznanski S Chew M Lee AJ Kolb M. M2-polarized and tumor-associated macrophages alter NK cell phenotype and function in a contact-dependent manner. J Leukoc Biol (2017) 101(1):285–95. doi: 10.1189/jlb.3A1215-552R 27493241

[46] Scavelli C Nico B Cirulli T Ria R Di Pietro G Mangieri D. Vasculogenic mimicry by bone marrow macrophages in patients with multiple myeloma. Oncogene (2008) 27(5):663–74. doi: 10.1038/sj.onc.1210691 17667938

[47] Zheng Y Cai Z Wang S Zhang X Qian J Hong S. Macrophages are an abundant component of myeloma microenvironment and protect myeloma cells from chemotherapy drug-induced apoptosis. Blood (2009) 114(17):3625–8. doi: 10.1182/blood-2009-05-220285 PMC2766678 19710503

[48] Opperman KS Vandyke K Psaltis PJ Noll JE Zannettino ACW. Macrophages in multiple myeloma: key roles and therapeutic strategies. Cancer Metastasis Rev (2021) 40(1):273–84. doi: 10.1007/s10555-020-09943-1 33404860

[49] Ribatti D Moschetta M Vacca A. Macrophages in multiple myeloma. Immunol Lett (2014) 161(2):241–4. doi: 10.1016/j.imlet.2013.12.010 24370642

[50] Beider K Bitner H Leiba M Gutwein O Koren-Michowitz M Ostrovsky O. Multiple myeloma cells recruit tumor-supportive macrophages through the CXCR4/CXCL12 axis and promote their polarization toward the M2 phenotype. Oncotarget (2014) 5(22):11283–96. doi: 10.18632/oncotarget.2207 PMC4294328 25526031

[51] Lanier LL. NK cell receptors. Annu Rev Immunol (1998) 16:359–93. doi: 10.1146/annurev.immunol.16.1.359 9597134

[52] Joncker NT Raulet DH. Regulation of NK cell responsiveness to achieve self-tolerance and maximal responses to diseased target cells. Immunol Rev (2008) 224:85–97. doi: 10.1111/j.1600-065X.2008.00658.x 18759922 PMC3017429

[53] Shifrin N Raulet DH Ardolino M. NK cell self tolerance, responsiveness and missing self recognition. Semin Immunol (2014) 26(2):138–44. doi: 10.1016/j.smim.2014.02.007 PMC3984600 24629893

[54] Pazina T MacFarlane A Bernabei L Dulaimi E Kotcher R Yam C. Alterations of NK cell phenotype in the disease course of multiple myeloma. Cancers (Basel) (2021) 13(2):226–48. doi: 10.3390/cancers13020226 PMC7827733 33435153

[55] Bernal M Garrido P Jimenez P Carretero R Almagro M Lopez P. Changes in activatory and inhibitory natural killer (NK) receptors may induce progression to multiple myeloma: implications for tumor evasion of T and NK cells. Hum Immunol (2009) 70(10):854–7. doi: 10.1016/j.humimm.2009.07.004 19580833

[56] Carbone E Neri P Mesuraca M Fulciniti MT Otsuki T Pende D. NKG2D, and natural cytotoxicity receptors regulate multiple myeloma cell recognition by natural killer cells. Blood (2005) 105(1):251–8. doi: 10.1182/blood-2004-04-1422 15328155

[57] Konjevic G Vuletic A Mirjacic Martinovic K Colovic N Colovic M Jurisic V. Decreased CD161 activating and increased CD158a inhibitory receptor expression on NK cells underlies impaired NK cell cytotoxicity in patients with multiple myeloma. J Clin Pathol (2016) 1136(10):1–8. doi: 10.1136/jclinpath-2016-203614 27083212

[58] Costello RT Boehrer A Sanchez C Mercier D Baier C Le Treut T. Differential expression of natural killer cell activating receptors in blood versus bone marrow in patients with monoclonal gammopathy. Immunology (2013) 139(3):338–41. doi: 10.1111/imm.12082 PMC3701180 23360454

[59] Gabrilovich DI. Myeloid-derived suppressor cells. Cancer Immunol Res (2017) 5(1):3–8. doi: 10.1158/2326-6066.CIR-16-0297 28052991 PMC5426480

[60] Law AMK Valdes-Mora F Gallego-Ortega D. Myeloid-derived suppressor cells as a therapeutic target for cancer. Cells (2020) 9(3):561–92. doi: 10.3390/cells9030561 PMC7140518 32121014

[61] Takizawa H Boettcher S Manz MG. Demand-adapted regulation of early hematopoiesis in infection and inflammation. Blood (2012) 119(13):2991–3002. doi: 10.1182/blood-2011-12-380113 22246037

[62] Gorgun GT Whitehill G Anderson JL Hideshima T Maguire C Laubach J. Tumor-promoting immune-suppressive myeloid-derived suppressor cells in the multiple myeloma microenvironment in humans. Blood (2013) 121(15):2975–87. doi: 10.1182/blood-2012-08-448548 PMC3624943 23321256

[63] Youn JI Nagaraj S Collazo M Gabrilovich DI. Subsets of myeloid-derived suppressor cells in tumor-bearing mice. J Immunol (2008) 181(8):5791–802. doi: 10.4049/jimmunol.181.8.5791 PMC2575748 18832739

[64] Botta C Gulla A Correale P Tagliaferri P Tassone P. Myeloid-derived suppressor cells in multiple myeloma: pre-clinical research and translational opportunities. Front Oncol (2014) 4:348. doi: 10.3389/fonc.2014.00348 25538892 PMC4258997

[65] Gao X Sui H Zhao S Gao X Su Y Qu P. Immunotherapy targeting myeloid-derived suppressor cells (MDSCs) in tumor microenvironment. Front Immunol (2020) 11:585214. doi: 10.3389/fimmu.2020.585214 33613512 PMC7889583

[66] Favaloro J Liyadipitiya T Brown R Yang S Suen H Woodland N. Myeloid derived suppressor cells are numerically, functionally and phenotypically different in patients with multiple myeloma. Leuk Lymphoma (2014) 55(12):2893–900. doi: 10.3109/10428194.2014.904511 24625328

[67] Ramachandran IR Martner A Pisklakova A Condamine T Chase T Vogl T. Myeloid-derived suppressor cells regulate growth of multiple myeloma by inhibiting T cells in bone marrow. J Immunol (2013) 190(7):3815–23. doi: 10.4049/jimmunol.1203373 PMC3608837 23460744

[68] Binsfeld M Muller J Lamour V De Veirman K De Raeve H Bellahcene A. Granulocytic myeloid-derived suppressor cells promote angiogenesis in the context of multiple myeloma. Oncotarget (2016) 7(25):37931–43. doi: 10.18632/oncotarget.9270 PMC5122361 27177328

[69] Petersson J Askman S Pettersson A Wichert S Hellmark T Johansson ACM. Bone marrow neutrophils of multiple myeloma patients exhibit myeloid-derived suppressor cell activity. J Immunol Res (2021) 2021:6344344. doi: 10.1155/2021/6344344 34414242 PMC8369183

[70] Romano A Parrinello NL Simeon V Puglisi F La Cava P Bellofiore C. High-density neutrophils in MGUS and multiple myeloma are dysfunctional and immune-suppressive due to increased STAT3 downstream signaling. Sci Rep (2020) 10(1):1983. doi: 10.1038/s41598-020-58859-x 32029833 PMC7005058

[71] Auffray C Sieweke MH Geissmann F. Blood monocytes: development, heterogeneity, and relationship with dendritic cells. Annu Rev Immunol (2009) 27:669–92. doi: 10.1146/annurev.immunol.021908.132557 19132917

[72] Zahran AM Nafady-Hego H Moeen SM Eltyb HA Wahman MM Nafady A. Higher proportion of non-classical and intermediate monocytes in newly diagnosed multiple myeloma patients in Egypt: A possible prognostic marker. Afr J Lab Med (2021) 10(1):129. doi: 10.4102/ajlm.v10i1.1296 34522628 PMC8424713

[73] Sponaas AM Moen SH Liabakk NB Feyzi E Holien T Kvam S. The proportion of CD16(+)CD14(dim) monocytes increases with tumor cell load in bone marrow of patients with multiple myeloma. Immun Inflammation Dis (2015) 3(2):94–102. doi: 10.1002/iid3.53 PMC4444152 26029369

[74] Rego EM Garcia AB Viana SR Falcao RP. Age-related changes of lymphocyte subsets in normal bone marrow biopsies. Cytometry (1998) 34(1):22–9. doi: 10.1002/(SICI)1097-0320(19980215)34:1<22::AID-CYTO4>3.0.CO;2-G 9580154

[75] San Miguel JF Gonzalez M Gascon A Moro MJ Hernandez JM Ortega F. Lymphoid subsets and prognostic factors in multiple myeloma. cooperative group for the study of monoclonal gammopathies. Br J Haematol (1992) 80(3):305–9. doi: 10.1111/j.1365-2141.1992.tb08137.x 1581210

[76] Quinn KM Kartikasari AER Cooke RE Koldej RM Ritchie DS Plebanski M. Impact of age-, cancer-, and treatment-driven inflammation on T cell function and immunotherapy. J Leukoc Biol (2020) 108(3):953–65. doi: 10.1002/JLB.5MR0520-466R 32678927

[77] Franceschi C Bonafe M Valensin S Olivieri F De Luca M Ottaviani E. Inflamm-aging. an evolutionary perspective on immunosenescence. Ann N Y Acad Sci (2000) 908:244–54. doi: 10.1111/j.1749-6632.2000.tb06651.x 10911963

[78] Zelle-Rieser C Thangavadivel S Biedermann R Brunner A Stoitzner P Willenbacher E. T Cells in multiple myeloma display features of exhaustion and senescence at the tumor site. J Hematol Oncol (2016) 9(1):116. doi: 10.1186/s13045-016-0345-3 27809856 PMC5093947

[79] Kuehn HS Ouyang W Lo B Deenick EK Niemela JE Avery DT. Immune dysregulation in human subjects with heterozygous germline mutations in CTLA4. Science (2014) 345(6204):1623–7. doi: 10.1126/science.1255904 PMC4371526 25213377

[80] Wang HY Wang RF. Regulatory T cells and cancer. Curr Opin Immunol (2007) 19(2):217–23. doi: 10.1016/j.coi.2007.02.004 17306521

[81] Alrasheed N Lee L Ghorani E Henry JY Conde L Chin M. Marrow-infiltrating regulatory T cells correlate with the presence of dysfunctional CD4(+)PD-1(+) cells and inferior survival in patients with newly diagnosed multiple myeloma. Clin Cancer Res (2020) 26(13):3443–54. doi: 10.1158/1078-0432.CCR-19-1714 32220887

[82] Bettelli E Carrier Y Gao W Korn T Strom TB Oukka M. Reciprocal developmental pathways for the generation of pathogenic effector TH17 and regulatory T cells. Nature (2006) 441(7090):235–8. doi: 10.1038/nature04753 16648838

[83] Ma T Zhang Y Zhou X Xie P Li J. A unique role of T helper 17 cells in different treatment stages of multiple myeloma. Clin Lymphoma Myeloma Leuk (2020) 20(3):190–7. doi: 10.1016/j.clml.2019.12.009 31980418

[84] Prabhala RH Pelluru D Fulciniti M Prabhala HK Nanjappa P Song W. Elevated IL-17 produced by TH17 cells promotes myeloma cell growth and inhibits immune function in multiple myeloma. Blood (2010) 115(26):5385–92. doi: 10.1182/blood-2009-10-246660 PMC2902136 20395418

[85] Suyani E Sucak GT Akyurek N Sahin S Baysal NA Yagci M. Tumor-associated macrophages as a prognostic parameter in multiple myeloma. Ann Hematol (2013) 92(5):669–77. doi: 10.1007/s00277-012-1652-6 23334187

[86] Wang H Hu WM Xia ZJ Liang Y Lu Y Lin SX. High numbers of CD163+ tumor-associated macrophages correlate with poor prognosis in multiple myeloma patients receiving bortezomib-based regimens. J Cancer (2019) 10(14):3239–45. doi: 10.7150/jca.30102 PMC6603386 31289595

[87] Sun M Xiao Q Wang X Yang C Chen C Tian X. Tumor-associated macrophages modulate angiogenesis and tumor growth in a xenograft mouse model of multiple myeloma. Leuk Res (2021) 110:106709. doi: 10.1016/j.leukres.2021.106709 34560409

[88] Velichinskii RA Streltsova MA Kust SA Sapozhnikov AM Kovalenko EI. The biological role and therapeutic potential of NK cells in hematological and solid tumors. Int J Mol Sci (2021) 22(21):11385–413. doi: 10.3390/ijms222111385 PMC8584101 34768814

[89] Russick J Torset C Hemery E Cremer I. NK cells in the tumor microenvironment: Prognostic and theranostic impact. Recent Adv trends Semin Immunol (2020) 48:101407. doi: 10.1016/j.smim.2020.101407 32900565

[90] Sivori S Pende D Quatrini L Pietra G Della Chiesa M Vacca P. NK cells and ILCs in tumor immunotherapy. Mol Aspects Med (2021) 80:100870. doi: 10.1016/j.mam.2020.100870 32800530

[91] Wu SY Fu T Jiang YZ Shao ZM. Natural killer cells in cancer biology and therapy. Mol Cancer (2020) 19(1):120. doi: 10.1158/1557-3125.HIPPO19-IA12 32762681 PMC7409673

[92] Frohn C Hoppner M Schlenke P Kirchner H Koritke P Luhm J. Anti-myeloma activity of natural killer lymphocytes. Br J Haematol (2002) 119(3):660–4. doi: 10.1046/j.1365-2141.2002.03879.x 12437641

[93] Vivier E Raulet DH Moretta A Caligiuri MA Zitvogel L Lanier LL. Innate or adaptive immunity? the example of natural killer cells. Science (2011) 331(6013):44–9. doi: 10.1126/science.1198687 PMC3089969 21212348

[94] Bottcher JP Bonavita E Chakravarty P Blees H Cabeza-Cabrerizo M Sammicheli S. NK cells stimulate recruitment of cDC1 into the tumor microenvironment promoting cancer immune control. Cell (2018) 172(5):1022–1037 e1014. doi: 10.1016/j.cell.2018.01.004 29429633 PMC5847168

[95] Fu B Tian Z Wei H. Subsets of human natural killer cells and their regulatory effects. Immunology (2014) 141(4):483–9. doi: 10.1111/imm.12224 PMC3956422 24303897

[96] Jurisic V Srdic T Konjevic G Markovic O Colovic M. Clinical stage-depending decrease of NK cell activity in multiple myeloma patients. Med Oncol (2007) 24(3):312–7. doi: 10.1007/s12032-007-0007-y 17873307

[97] Campbell KS Cohen AD Pazina T. Mechanisms of NK cell activation and clinical activity of the therapeutic SLAMF7 antibody, elotuzumab in multiple myeloma. Front Immunol (2018) 9:2551. doi: 10.3389/fimmu.2018.02551 30455698 PMC6230619

[98] Orrantia A Terren I Astarloa-Pando G Gonzalez C Uranga A Mateos-Mazon JJ. NK cell reconstitution after autologous hematopoietic stem cell transplantation: Association between NK cell maturation stage and outcome in multiple myeloma. Front Immunol (2021) 12:748207. doi: 10.3389/fimmu.2021.748207 34675932 PMC8524090

[99] Xu Y Zhang X Liu H Zhao P Chen Y Luo Y. Mesenchymal stromal cells enhance the suppressive effects ofmyeloid-derived suppressor cells of multiple myeloma. Leuk Lymphoma (2017) 58(11):2668–76. doi: 10.1080/10428194.2017.1298753 28317413

[100] De Veirman K Van Ginderachter JA Lub S De Beule N Thielemans K Bautmans I. Multiple myeloma induces mcl-1 expression and survival of myeloid-derived suppressor cells. Oncotarget (2015) 6(12):10532–47. doi: 10.18632/oncotarget.3300 PMC4496373 25871384

[101] Mozaffari F Hansson L Kiaii S Ju X Rossmann ED Rabbani H. Signalling molecules and cytokine production in T cells of multiple myeloma-increased abnormalities with advancing stage. Br J Haematol (2004) 124(3):315–24. doi: 10.1046/j.1365-2141.2003.04789.x 14717778

[102] Prabhala RH Neri P Bae JE Tassone P Shammas MA Allam CK. Dysfunctional T regulatory cells in multiple myeloma. Blood (2006) 107(1):301–4. doi: 10.1182/blood-2005-08-3101 PMC1895365 16150935

[103] Dahlhoff J Manz H Steinfatt T Delgado-Tascon J Seebacher E Schneider T. Transient regulatory T-cell targeting triggers immune control of multiple myeloma and prevents disease progression. Leukemia (2022) 36(3):790–800. doi: 10.1038/s41375-021-01422-y 34584204 PMC8885410

[104] Joshua DE Vuckovic S Favaloro J Lau KHA Yang S Bryant CE. Treg and oligoclonal expansion of terminal effector CD8(+) T cell as key players in multiple myeloma. Front Immunol (2021) 12:620596. doi: 10.3389/fimmu.2021.620596 33708212 PMC7940512

[105] Favaloro J Brown R Aklilu E Yang S Suen H Hart D. *et al.* myeloma skews regulatory T and pro-inflammatory T helper 17 cell balance in favor of a suppressive state. Leuk Lymphoma (2014) 55(5):1090–8. doi: 10.3109/10428194.2013.825905 23865833

[106] Lad D Huang Q Hoeppli R Garcia R Xu L Levings M. Evaluating the role of tregs in the progression of multiple myeloma. Leuk Lymphoma (2019) 60(9):2134–42. doi: 10.1080/10428194.2019.1579324 30773086

[107] Sharma A Khan R Joshi S Kumar L Sharma M. Dysregulation in T helper 1/T helper 2 cytokine ratios in patients with multiple myeloma. Leuk Lymphoma (2010) 51(5):920–7. doi: 10.3109/10428191003699563 20367137

[108] Rosser EC Mauri C. Regulatory b cells: origin, phenotype, and function. Immunity (2015) 42(4):607–12. doi: 10.1016/j.immuni.2015.04.005 25902480

[109] Alhabbab RY Nova-Lamperti E Aravena O Burton HM Lechler RI Dorling A. Regulatory b cells: Development, phenotypes, functions, and role in transplantation. Immunol Rev (2019) 292(1):164–79. doi: 10.1111/imr.12800 31559645

[110] Tamura H. Immunopathogenesis and immunotherapy of multiple myeloma. Int J Hematol (2018) 107(3):278–85. doi: 10.1007/s12185-018-2405-7 29368256

[111] Zhang L Tai YT Ho M Xing L Chauhan D Gang A. Regulatory b cell-myeloma cell interaction confers immunosuppression and promotes their survival in the bone marrow milieu. Blood Cancer J (2017) 7(3):e547. doi: 10.1038/bcj.2017.24 28338671 PMC5380908

[112] Zou Z Guo T Cui J Tang W Li Y Wang F. Real-world data combined with studies on regulatory b cells for newly diagnosed multiple myeloma from a tertiary referral hospital in south-Western China. J Cancer (2021) 12(9):2633–42. doi: 10.7150/jca.53209 PMC8040702 33854623

[113] Bartosinska J Purkot J Karczmarczyk A Chojnacki M Zaleska J Wlasiuk P. Differential function of a novel population of the CD19+CD24hiCD38hi bregs in psoriasis and multiple myeloma. Cells (2021) 10(2):411–21. doi: 10.3390/cells10020411 PMC7920433 33669402

[114] Cui J Zou Z Duan J Tang W Li Y Zhang L. Predictive values of PET/CT in combination with regulatory b cells for therapeutic response and survival in contemporary patients with newly diagnosed multiple myeloma. Front Immunol (2021) 12:671904. doi: 10.3389/fimmu.2021.671904 34489930 PMC8417409

[115] Podar K Chauhan D Anderson KC. Bone marrow microenvironment and the identification of new targets for myeloma therapy. Leukemia (2009) 23(1):10–24. doi: 10.1038/leu.2008.259 18843284 PMC3418600

[116] Akhtar S Ali TA Faiyaz A Khan OS Raza SS Kulinski M. Cytokine-mediated dysregulation of signaling pathways in the pathogenesis of multiple myeloma. Int J Mol Sci (2020) 21(14):5002–26. doi: 10.3390/ijms21145002 PMC7403981 32679860

[117] Giannakoulas N Ntanasis-Stathopoulos I Terpos E. The role of marrow microenvironment in the growth and development of malignant plasma cells in multiple myeloma. Int J Mol Sci (2021) 22(9):4462–80. doi: 10.3390/ijms22094462 PMC8123209 33923357

[118] Harmer D Falank C Reagan MR. Interleukin-6 interweaves the bone marrow microenvironment, bone loss, and multiple myeloma. Front Endocrinol (Lausanne) (2018) 9:788. doi: 10.3389/fendo.2018.00788 30671025 PMC6333051

[119] Duan LJ Li C Yang RY. [Values of detecting the levels of beta2-MG, TNF-alpha, CRP, IL-6 in the patients with multiple myeloma]. Zhongguo Shi Yan Xue Ye Xue Za Zhi (2015) 23(5):1362–5. doi: 10.7534/j.issn.1009-2137.2015.05.026 26524038

[120] Ballester OF Moscinski LC Lyman GH Chaney JV Saba HI Spiers AS. High levels of interleukin-6 are associated with low tumor burden and low growth fraction in multiple myeloma. Blood (1994) 83(7):1903–8. doi: 10.1182/blood.V83.7.1903.1903 8142657

[121] Frassanito MA Cusmai A Iodice G Dammacco F. Autocrine interleukin-6 production and highly malignant multiple myeloma: relation with resistance to drug-induced apoptosis. Blood (2001) 97(2):483–9. doi: 10.1182/blood.V97.2.483 11154226

[122] Sfiridaki A Miyakis S Tsirakis G Alegakis A Passam AM Kandidaki E. Systemic levels of interleukin-6 and matrix metalloproteinase-9 in patients with multiple myeloma may be useful as prognostic indexes of bone disease. Clin Chem Lab Med (2005) 43(9):934–8. doi: 10.1515/CCLM.2005.160 16176173

[123] Abildgaard N Glerup H Rungby J Bendix-Hansen K Kassem M Brixen K. Biochemical markers of bone metabolism reflect osteoclastic and osteoblastic activity in multiple myeloma. Eur J Haematol (2000) 64(2):121–9. doi: 10.1034/j.1600-0609.2000.90074.x 10997332

[124] Saraiva M Vieira P O'Garra A. Biology and therapeutic potential of interleukin-10. J Exp Med (2020) 217(1):e20190418. doi: 10.1084/jem.20190418 31611251 PMC7037253

[125] Ameglio F Alvino S Trento E Marcucci M Pimpinelli F Tong A. Serum interleukin-10 levels in patients affected with multiple-myeloma - correlation with the monoclonal component and disease progression. Int J Oncol (1995) 6(6):1189–92. doi: 10.3892/ijo.6.6.1189 21556657

[126] Wang H Wang L Chi PD Wang WD Chen XQ Geng QR. High level of interleukin-10 in serum predicts poor prognosis in multiple myeloma. Br J Cancer (2016) 114(4):463–8. doi: 10.1038/bjc.2016.11 PMC4815778 26882069

[127] Alexandrakis MG Goulidaki N Pappa CA Boula A Psarakis F Neonakis I. Interleukin-10 induces both plasma cell proliferation and angiogenesis in multiple myeloma. Pathol Oncol Res (2015) 21(4):929–34. doi: 10.1007/s12253-015-9921-z 25743259

[128] Minnie SA Kuns RD Gartlan KH Zhang P Wilkinson AN Samson L. Myeloma escape after stem cell transplantation is a consequence of T-cell exhaustion and is prevented by TIGIT blockade. Blood (2018) 132(16):1675–88. doi: 10.1182/blood-2018-01-825240 30154111

[129] Isufi I Seetharam M Zhou L Sohal D Opalinska J Pahanish P. Transforming growth factor-beta signaling in normal and malignant hematopoiesis. J Interferon Cytokine Res (2007) 27(7):543–52. doi: 10.1089/jir.2007.0009 17651015

[130] Dong M Blobe GC. Role of transforming growth factor-beta in hematologic malignancies. Blood (2006) 107(12):4589–96. doi: 10.1182/blood-2005-10-4169 PMC1895802 16484590

[131] Urashima M Ogata A Chauhan D Hatziyanni M Vidriales MB Dedera DA. Transforming growth factor-beta1: differential effects on multiple myeloma versus normal b cells. Blood (1996) 87(5):1928–38. doi: 10.1182/blood.V87.5.1928.1928 8634441

[132] Kyrtsonis MC Repa C Dedoussis GV Mouzaki A Simeonidis A Stamatelou M. Serum transforming growth factor-beta 1 is related to the degree of immunoparesis in patients with multiple myeloma. Med Oncol (1998) 15(2):124–8. doi: 10.1007/BF02989591 9789221

[133] Lambert KE Huang H Mythreye K Blobe GC. The type III transforming growth factor-beta receptor inhibits proliferation, migration, and adhesion in human myeloma cells. Mol Biol Cell (2011) 22(9):1463–72. doi: 10.1091/mbc.e10-11-0877 PMC3084669 21411633

[134] Lu A Pallero MA Lei W Hong H Yang Y Suto MJ. Inhibition of transforming growth factor-beta activation diminishes tumor progression and osteolytic bone disease in mouse models of multiple myeloma. Am J Pathol (2016) 186(3):678–90. doi: 10.1016/j.ajpath.2015.11.003 PMC4816696 26801735

[135] Green AC Lath D Hudson K Walkley B Down JM Owen R. TGFbeta inhibition stimulates collagen maturation to enhance bone repair and fracture resistance in a murine myeloma model. J Bone Miner Res (2019) 34(12):2311–26. doi: 10.1002/jbmr.3859 31442332

[136] Takeuchi K Abe M Hiasa M Oda A Amou H Kido S. Tgf-beta inhibition restores terminal osteoblast differentiation to suppress myeloma growth. PLoS One (2010) 5(3):e9870. doi: 10.1371/journal.pone.0009870 20360846 PMC2845613

[137] Alexandrakis MG Passam FH Boula A Christophoridou A Aloizos G Roussou P. Relationship between circulating serum soluble interleukin-6 receptor and the angiogenic cytokines basic fibroblast growth factor and vascular endothelial growth factor in multiple myeloma. Ann Hematol (2003) 82(1):19–23. doi: 10.1007/s00277-002-0558-0 12574959

[138] Palta A Kaur M Tahlan A Dimri K. Evaluation of angiogenesis in multiple myeloma by VEGF immunoexpression and microvessel density. J Lab Phys (2020) 12(1):38–43. doi: 10.1055/s-0040-1714933 PMC7419170 32792792

[139] Brito AB Lourenco GJ Oliveira GB De Souza CA Vassallo J Lima CS. Associations of VEGF and VEGFR2 polymorphisms with increased risk and aggressiveness of multiple myeloma. Ann Hematol (2014) 93(8):1363–9. doi: 10.1007/s00277-014-2062-8 24687381

[140] Shimon I Shpilberg O. The insulin-like growth factor system in regulation of normal and malignant hematopoiesis. Leuk Res (1995) 19(4):233–40. doi: 10.1016/0145-2126(94)00133-U 7538616

[141] Vishwamitra D George SK Shi P Kaseb AO Amin HM. Type I insulin-like growth factor receptor signaling in hematological malignancies. Oncotarget (2017) 8(1):1814–44. doi: 10.18632/oncotarget.12123 PMC5352101 27661006

[142] Qiang YW Yao L Tosato G Rudikoff S. Insulin-like growth factor I induces migration and invasion of human multiple myeloma cells. Blood (2004) 103(1):301–8. doi: 10.1182/blood-2003-06-2066 14504085

[143] Peng Y Li F Zhang P Wang X Shen Y Feng Y. IGF-1 promotes multiple myeloma progression through PI3K/Akt-mediated epithelial-mesenchymal transition. Life Sci (2020) 249:117503. doi: 10.1016/j.lfs.2020.117503 32142767

[144] Georgii-Hemming P Wiklund HJ Ljunggren O Nilsson K. Insulin-like growth factor I is a growth and survival factor in human multiple myeloma cell lines. Blood (1996) 88(6):2250–8. doi: 10.1182/blood.V88.6.2250.bloodjournal8862250 8822946

[145] Standal T Borset M Lenhoff S Wisloff F Stordal B Sundan A. Serum insulinlike growth factor is not elevated in patients with multiple myeloma but is still a prognostic factor. Blood (2002) 100(12):3925–9. doi: 10.1182/blood-2002-05-1406 12393395

[146] Yang P Hu Y Zhou Q. The CXCL12-CXCR4 signaling axis plays a key role in cancer metastasis and is a potential target for developing novel therapeutics against metastatic cancer. Curr Med Chem (2020) 27(33):5543–61. doi: 10.2174/0929867326666191113113110 31724498

[147] Peled A Klein S Beider K Burger JA Abraham M. Role of CXCL12 and CXCR4 in the pathogenesis of hematological malignancies. Cytokine (2018) 109:11–6. doi: 10.1016/j.cyto.2018.02.020 29903571

[148] Trentin L Miorin M Facco M Baesso I Carraro S Cabrelle A. Multiple myeloma plasma cells show different chemokine receptor profiles at sites of disease activity. Br J Haematol (2007) 138(5):594–602. doi: 10.1111/j.1365-2141.2007.06686.x 17686053

[149] Vande Broek I Leleu X Schots R Facon T Vanderkerken K Van Camp B. Clinical significance of chemokine receptor (CCR1, CCR2 and CXCR4) expression in human myeloma cells: the association with disease activity and survival. Haematologica (2006) 91(2):200–6. doi: 10.1111/j.1365-2648.2008.04906.x 16461304

[150] Bao L Lai Y Liu Y Qin Y Zhao X Lu X. CXCR4 is a good survival prognostic indicator in multiple myeloma patients. Leuk Res (2013) 37(9):1083–8. doi: 10.1016/j.leukres.2013.06.002 23849988

[151] Zannettino AC Farrugia AN Kortesidis A Manavis J To LB Martin SK. Elevated serum levels of stromal-derived factor-1alpha are associated with increased osteoclast activity and osteolytic bone disease in multiple myeloma patients. Cancer Res (2005) 65(5):1700–9. doi: 10.1158/0008-5472.CAN-04-1687 15753365

[152] Martin SK Dewar AL Farrugia AN Horvath N Gronthos S To LB. Tumor angiogenesis is associated with plasma levels of stromal-derived factor-1alpha in patients with multiple myeloma. Clin Cancer Res (2006) 12(23):6973–7. doi: 10.1158/1078-0432.CCR-06-0323 17145816

[153] Giallongo C Dulcamare I Tibullo D Del Fabro V Vicario N Parrinello N. CXCL12/CXCR4 axis supports mitochondrial trafficking in tumor myeloma microenvironment. Oncogenesis (2022) 11(1):6. doi: 10.1038/s41389-022-00380-z 35064098 PMC8782911

[154] Liu Y Liang HM Lv YQ Tang SM Cheng P. Blockade of SDF-1/CXCR4 reduces adhesion-mediated chemoresistance of multiple myeloma cells *via* interacting with interleukin-6. J Cell Physiol (2019) 234(11):19702–14. doi: 10.1002/jcp.28570 30953364

[155] Alsayed Y Ngo H Runnels J Leleu X Singha UK Pitsillides CM. Mechanisms of regulation of CXCR4/SDF-1 (CXCL12)-dependent migration and homing in multiple myeloma. Blood (2007) 109(7):2708–17. doi: 10.1182/blood-2006-07-035857 PMC1852222 17119115

[156] Diamond P Labrinidis A Martin SK Farrugia AN Gronthos S To LB. Targeted disruption of the CXCL12/CXCR4 axis inhibits osteolysis in a murine model of myeloma-associated bone loss. J Bone Miner Res (2009) 24(7):1150–61. doi: 10.1359/jbmr.090210 19335218

